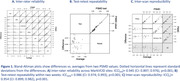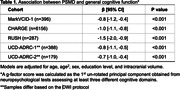# Instrumental and biological validation of PSMD as a VCID biomarker

**DOI:** 10.1002/alz.086590

**Published:** 2025-01-09

**Authors:** Claudia L Satizabal, Alexa S Beiser, Alison M. Luckey, Rebecca Bernal, Saptaparni Ghosh, Chen‐Pin Wang, Zhiguang Li, Djass Mbangdadji, Angel G Velarde, Hector A Trevino, Monica Goss, Laura J. Hillmer, Christopher E. Bauer, Adam M. Staffaroni, Lara Stables, Marilyn S. Albert, Jayandra J. Himali, Thomas H. Mosley, Lars Forsberg, Vilmundur Gudnason, Baljeet Singh, Herpreet Singh, Kristin Schwab, Joel H. Kramer, Gary A. Rosenberg, Karl G Helmer, Steven M. Greenberg, Mohamad Habes, Danny JJ Wang, Brian T Gold, Hanzhang Lu, Caprihan Arvind, Myriam Fornage, Lenore J. Launer, Konstantinos Arfanakis, Charles Decarli, Pauline Maillard, Sudha Seshadri

**Affiliations:** ^1^ The University of Texas Health Science Center at San Antonio, San Antonio, TX USA; ^2^ Boston University Chobanian & Avedisian School of Medicine, Boston, MA USA; ^3^ Glenn Biggs Institute for Alzheimer’s & Neurodegenerative Diseases, University of Texas Health Science Center, San Antonio, TX USA; ^4^ Glenn Biggs Institute for Alzheimer's & Neurodegenerative Diseases, UT Health San Antonio, San Antonio, TX USA; ^5^ Glenn Biggs Institute for Alzheimer’s & Neurodegenerative Diseases, San Antonio, TX USA; ^6^ National Institute on Aging, Baltimore, MD USA; ^7^ Glenn Biggs Institute for Alzheimer's & Neurodegenerative Diseases, University of Texas Health San Antonio, San Antonio, TX USA; ^8^ University of Texas Health San Antonio, San Antonio, TX USA; ^9^ University of New Mexico, Albuquerque, NM USA; ^10^ University of Kentucky, Lexington, KY USA; ^11^ Memory and Aging Center, UCSF Weill Institute for Neurosciences, University of California, San Francisco, San Francisco, CA USA; ^12^ Johns Hopkins University, Baltimore, MD USA; ^13^ University of Mississippi Medical Center, Jackson, MS USA; ^14^ Uppsala University, Uppsala Sweden; ^15^ Icelandic Heart Association, Kopavogur Iceland; ^16^ University of California, Davis, Davis, CA USA; ^17^ Massachusetts General Hospital, Boston, MA USA; ^18^ Athinoula A. Martinos Center for Biomedical Imaging, Massachusetts General Hospital, Harvard Medical School, Charlestown, MA USA; ^19^ Glenn Biggs Institute for Alzheimer’s & Neurodegenerative Diseases, University of Texas Health Sciences Center at San Antonio, San Antonio, TX USA; ^20^ Laboratory of FMRI Technology (LOFT), Mark & Mary Stevens Neuroimaging and Informatics Institute, University of Southern California, Los Angeles, CA USA; ^21^ Johns Hopkins University School of Medicine, Baltimore, MD USA; ^22^ The Mind Research Network, Albuquerque, NM USA; ^23^ The University of Texas Health Science Center at Houston, Houston, TX USA; ^24^ Brown Foundation Institute of Molecular Medicine, McGovern Medical School; School of Public Health, The University of Texas Health Science Center, Houston, TX USA; ^25^ Department of Diagnostic Radiology and Nuclear Medicine, Rush UniversityRush University Medical Center, Chicago, IL USA; ^26^ IDeA Laboratory, Department of Neurology, UC Davis, Davis, CA USA; ^27^ Alzheimer's Disease Research Center, University of California Davis, Sacramento, CA USA

## Abstract

**Background:**

Peak‐width of skeletonized mean diffusivity (PSMD) is an emerging biomarker of cerebral small vessel disease (cSVD)‐related vascular contributions to cognitive impairment and dementia (VCID). Higher PSMD values reflect greater white matter microstructural damage, and prior research has related PSMD to sporadic and monogenic forms of cSVD and worse cognitive function. Therefore, we proposed PSMD as a risk stratification biomarker for VCID. This study aimed to perform a rigorous instrumental and biological validation for PSMD in the MarkVCID‐1 consortium.

**Method:**

Methods to derive PSMD were packaged in a kit containing a protocol, scripts, and instructions. The instrumental validation included a pre‐specified plan to assess inter‐rater reliability, test‐retest repeatability, and inter‐scanner reproducibility among MarkVCID‐1 participants aged 53‐78 years across the spectrum of cSVD. We used intra‐class correlations for absolute agreement (ICC_AA_) and consistency (ICC_C_) to evaluate results, with ICC>0.07 as the pre‐specified goal.

The biological validation was performed on 7,289 participants of diverse ages and racial/ethnic backgrounds from MarkVCID‐1 and population‐based cohorts from CHARGE, RUSH, and UCD‐ADRC. All sites derived a composite measure of general cognitive function using neuropsychological tests assessing distinct cognitive domains. Finally, we used linear regression models to assess the association between log‐PSMD and general cognition adjusting for age, age^2^, sex, and education.

**Result:**

Our instrumental validation results (Figure 1) showed excellent reliability between raters from seven sites (overall ICC_AA_=0.945, P<0.001), agreement between test and retest measurements obtained within two weeks (ICC_AA_=0.986, P<0.001), and reproducibility across Philips Achieva, Siemens Prisma, and Siemens Trio scanners (ICC_C_= 0.954, P<0.001).

In the biological validation, higher PSMD values were associated with lower general cognitive function in MarkVCID‐1 (Beta=‐0.82, P<0.001), and these findings were replicated across the CHARGE, RUSH, and UCD‐ADRC cohorts (Table 1).

**Conclusion:**

Our rigorous instrumental validation study showed excellent inter‐rater reliability, test‐retest repeatability, and inter‐scanner reproducibility for the PSMD kit. We further observed strong associations between higher PSMD values and poorer cognitive function across diverse samples in the biological validation. Taken together, our findings support using PSMD as a robust risk stratification biomarker in multi‐site clinical trials of VCID. Additional longitudinal validation studies for PSMD are underway in MarkVCID‐2.